# Fractured Inferior Pubic Ramus with Ipsilateral Total Hip Replacement: A Case Report and Review of the Literature

**DOI:** 10.1155/2013/674732

**Published:** 2013-08-12

**Authors:** Sarkhell Radha, Michael Shenouda, Alexandra Hazlerigg, Sujith Konan, Alison Hulme

**Affiliations:** Department of Trauma & Orthopaedics, Chelsea & Westminster Hospital, 369 Fulham Road, London SW10 9NH, UK

## Abstract

Pubic rami fractures are common. They are associated with significant morbidity and mortality. These fractures are usually classified as stable injuries and traditionally receive limited orthopaedic input. Management typically involves hospital admission and early input from physiotherapists and occupational therapists. Early mobilisation is advocated as a central part of managing these patients, with emphasis on secondary prevention. We report a case diagnosed as minimally displaced inferior pubic ramus fracture in a patient with an ipsilateral total hip replacement (THR). The patient was mobilised early and despite analgesia continued to complain of groin pain. Repeat radiographs showed a fracture of the acetabulum with displacement of the acetabular component of the hip replacement. We advocate early orthopaedic input for all pubic rami fractures, particularly in patients with hip arthroplasty, and thorough investigation including a CT scan of the pelvis to exclude acetabular extension prior to mobilisation.

## 1. Introduction

Pubic rami fractures are the most common pelvic fracture pattern, with an estimated incidence of 25.6/100000 per year in individuals over 60 years [1]. This figure is predicted to increase with an aging population [2]. Patients can have significant morbidity and mortality associated with a pubic ramus fracture, with one-year mortality estimated at 13% and 5-year survival at 45.6% [1]. Poor prognostic indicators include an older age and presence of dementia [1].

In practice, the discovery of a pubic ramus fracture would not normally generate significant concern [3]. The patient is usually discharged with adequate analgesia and started on an active early mobilisation program [3]. There is currently a debate regarding which speciality is most appropriate to care for patients with pubic rami fractures. A study performed at the Royal Infirmary of Edinburgh reported that 80% of their patients with pelvic fractures did not require orthopaedic input [1]. The authors advocated that all patients with an isolated pubic ramus fracture should be admitted to the geriatric unit with orthopaedic input limited only for those cases with additional fractures [1].

We report a case of inferior pubic ramus fracture in a patient with a total hip arthroplasty. In this case, early mobilisation had a detrimental effect on the patient's outcome.

## 2. Case Presentation

**Figure 1:**
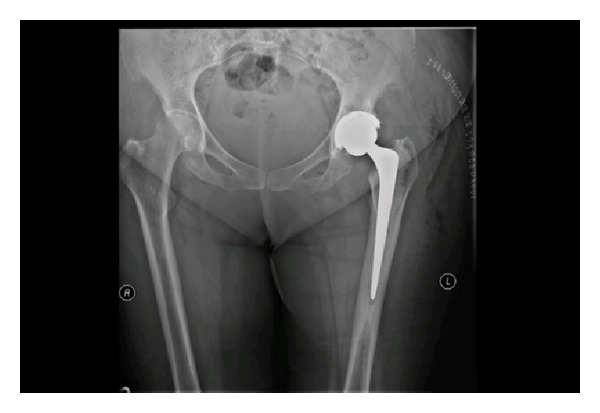
Plain radiograph taken on day 1 after initial left total hip arthroplasty.

**Figure 2:**
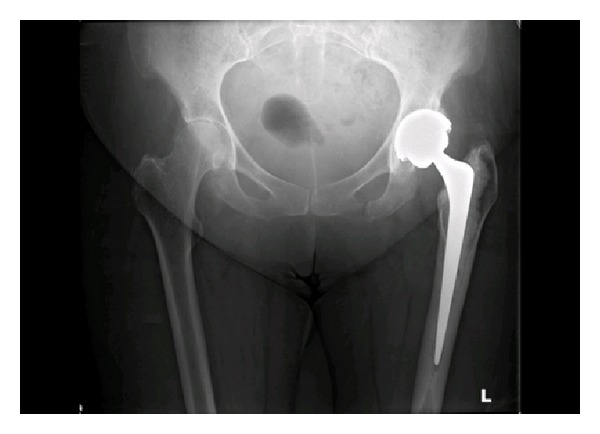
Plain radiograph taken following initial presentation with a fall, showing a fracture of the left inferior pubic ramus.

A 64-year-old lady with past medical history of learning difficulties and osteoarthritis presented to our emergency department following a mechanical trip and fall landing onto her left side. Detailed history and examination revealed pain and tenderness in the left groin over the inferior pubic ramus. The patient had pain on weight-bearing on the left side. She had previously undergone a left THR seven months earlier (Figure 1) and had been ambulating well without any problems since. Plain pelvic radiographs taken on this admission revealed only an isolated minimally displaced fracture of the left inferior pubic ramus (Figure 2). The patient was treated with analgesia and early mobilisation within the range of comfort with crutches. No followup was arranged and the patient was not discussed with the orthopaedic team.

**Figure 3:**
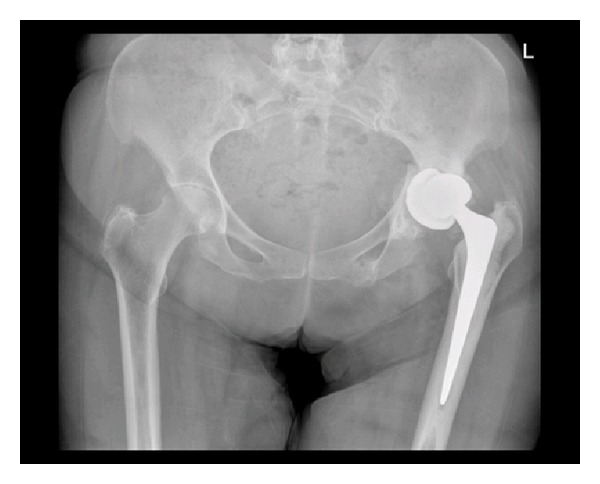
Pelvic radiograph taken after presentation with ongoing groin pain, showing fracture of the medial wall of the acetabulum with displacement of the acetabular component of the total hip replacement.

**Figure 4:**
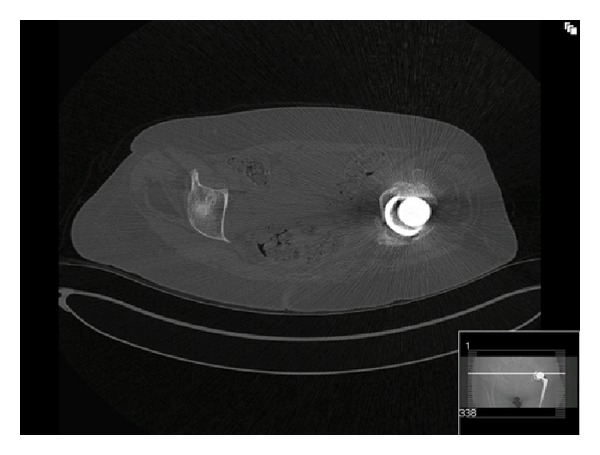
Pelvic CT scan confirming the acetabular fracture and displacement of acetabular component of the total hip replacement.

Three months later, she was still complaining of left hip pain and difficulty mobilising while using crutches. Further pelvic radiographs were performed and confirmed a fracture of the medial wall of the acetabulum with displacement of the acetabular component of the total hip replacement (Figures 3 and 4). There was no history of trauma between the two admissions. Subsequently, she was transferred to a specialist centre for fixation of this complex periprosthetic pelvic fracture.

## 3. Discussion

Pubic ramus fractures are common, and these are regarded as stable pelvic injuries [4]. Stress fracture of the pubic ramus following total hip arthroplasty (THA) has been reported in the literature. These cases presented several years after THA with no history of trauma. The authors recommended protective weight-bearing with successful outcomes [4].

Insufficiency fracture around the acetabular component of a THA is an unusual injury and this has been reported in the literature [5]. Kanaji et al. reported three cases of insufficiency fracture involving the medial wall of acetabulum following ipsilateral THR [6]. 

There have also been cases reported in the literature of occult acetabular fractures, both in native hips and following THA. Kakar et al. [7] reported three cases of occult acetabular fractures following a fall, identified only after further imaging due to ongoing discomfort and difficulty walking. There was an ipsilateral THA present in one of these patients. Guerado et al. [8] reported three further cases of occult acetabular fractures, again with delayed diagnosis. In this case series, none of the patients had a history of trauma, and all three had previous ipsilateral hip surgery with implants (intramedullary fixation for fractured proximal femur in two patients and a THA in the other).

Pubic ramus fractures can occur intra- and post operatively. In the majority of postoperative cases, these fractures occur as a result of osteolysis around the acetabular component which may take time to develop following primary surgery. These injuries are quite complex and in most cases require complex revision surgery [9].

In our case, the fracture of the inferior pubic ramus was identified on initial plain film radiographs, which had been reported by a consultant radiologist. The patient was encouraged to mobilise early and offered analgesia following the initial radiographic findings. Unfortunately, the presence of a periprosthetic acetabular fracture in relation to the THA was not recognised. 

An isolated inferior pubic ramus fracture is regarded as a stable injury and has much better functional outcome as compared to superior pubic rami fractures [10]. This assumption of a “stable injury” in our case led to the patient being actively mobilised with early discharge home following adequate pain control. It was only after representation with persistent groin pain that repeat radiographs and a subsequent CT were carried out and the extent of the injury was fully recognised, confirming the concomitant acetabular fracture.

This case represents a possible undiagnosed acetabular fracture missed on initial radiographic imaging. The pelvis is a ring structure, and if there is a fracture in one area, there may be a fracture or dislocation in another portion of the ring. Therefore, isolated fractures of the pubic ramus may well indicate further injuries elsewhere along the pelvic ring and should not be underestimated. This case highlights the need for specialist input in all pubic ramus fractures.

Early assessment for potential mobility and subsequent fast-track discharge planning may be appropriate for some patients with isolated pubic rami fractures. However, it is vital to appropriately choose patients for this so as to avoid the complications of missing more extensive fractures. Patients with isolated pubic ramus fractures should be evaluated carefully, looking for clinical findings, mechanism of injury, and pelvic or sacral tenderness. In the presence of clinical suspicion, patients should be investigated further with a CT scan. We believe that if our patient was investigated with CT scanning earlier, the fracture could have been identified and a period of non-weight-bearing advised. This may have prevented the acetabular fracture from displacement.

As acetabular fractures in the elderly may also occur with minimal or no trauma, and even in patients without previous ipsilateral hip surgery, a CT scan may also be indicated in patients reporting ongoing groin pain but with normal plain radiographs. Early mobilisation and a rapid rehabilitation programme should only be instituted when additional bony injuries have been excluded.

## 4. Conclusion

Pubic rami fractures may occur in isolation or be associated with other pelvic bony injuries. In the presence of clinical suspicion, further imaging in the form of a CT scan is recommended in order to confirm the presence or absence of other injuries. One should have a low threshold for further investigations, particularly in patients with previous hip arthroplasty. Only after exclusion of additional injury should early active mobilisation and rehabilitation begin.
